# Utility of electronic patient records in primary care for stroke secondary prevention trials

**DOI:** 10.1186/1471-2458-11-86

**Published:** 2011-02-07

**Authors:** Alex Dregan, Michael A Toschke, Charles D Wolfe, Anthony Rudd, Mark Ashworth, Martin C Gulliford

**Affiliations:** 1 Division of Health and Social Care Research, King's College London, Capital House, Weston Street, London, UK; 2 Department of Medical Informatics, Biometry and Epidemiology (IBE) and Munich Centre of Health Sciences (MC-Health), Ludwig-Maximilians University of Munich, Munich, Germany

## Abstract

**Background:**

This study aimed to inform the design of a pragmatic trial of stroke prevention in primary care by evaluating data recorded in electronic patient records (EPRs) as potential outcome measures. The study also evaluated achievement of recommended standards of care; variation between family practices; and changes in risk factor values from before to after stroke.

**Methods:**

Data from the UK General Practice Research Database (GPRD) were analysed for 22,730 participants with an index first stroke between 2003 and 2006 from 414 family practices. For each subject, the EPR was evaluated for the 12 months before and after stroke. Measures relevant to stroke secondary prevention were analysed including blood pressure (BP), cholesterol, smoking, alcohol use, body mass index (BMI), atrial fibrillation, utilisation of antihypertensive, antiplatelet and cholesterol lowering drugs. Intraclass correlation coefficients (ICC) were estimated by family practice. Random effects models were fitted to evaluate changes in risk factor values over time.

**Results:**

In the 12 months following stroke, BP was recorded for 90%, cholesterol for 70% and body mass index (BMI) for 47%. ICCs by family practice ranged from 0.02 for BP and BMI to 0.05 for LDL and HDL cholesterol. For subjects with records available both before and after stroke, the mean reductions from before to after stroke were: mean systolic BP, 6.02 mm Hg; diastolic BP, 2.78 mm Hg; total cholesterol, 0.60 mmol/l; BMI, 0.34 Kg/m^2^. There was an absolute reduction in smokers of 5% and heavy drinkers of 4%. The proportion of stroke patients within the recommended guidelines varied from less than a third (29%) for systolic BP, just over half for BMI (54%), and over 90% (92%) on alcohol consumption.

**Conclusions:**

Electronic patient records have potential for evaluation of outcomes in pragmatic trials of stroke secondary prevention. Stroke prevention interventions in primary care remain suboptimal but important reductions in vascular risk factor values were observed following stroke. Better recording of lifestyle factors in the GPRD has the potential to expand the scope of the GPRD for health care research and practice.

## Background

Individuals who survive stroke are at increased risk of recurrent stroke [1]. The risk of recurrence is highest during the first year following a stroke but remains elevated for at least 10 years [2,3]. Clinical practice recommendations for secondary prevention of stroke, including the UK Intercollegiate Stroke Working Party (ICSWP) guidelines [4] and the American Heart Association (AHA) guidelines, [5] emphasise the requirement to intervene to reduce the risk of further strokes, and other cardiovascular events, by targeting major modifiable vascular risk factors including elevated blood pressure (BP), and high cholesterol levels, as well as implementing antiplatelet therapy and lifestyle change when appropriate. These recommendations draw on evidence from meta-analyses of randomised controlled trials and other studies that provide evidence of the protective role of antiplatelet, cholesterol lowering and antihypertensive therapy against the risk of recurrent strokes and other vascular events [6-12].

Few nationally representative, population-based studies of stroke secondary prevention, and changes in vascular risk factor values following stroke, have been reported. The present study therefore aimed to evaluate the implementation of stroke secondary prevention in primary care by using electronic patient records (EPR) from a large population. The analyses had the additional purpose of informing the design of a cluster randomised trial of computerised decision support. The development of the intervention for the study has been reported elsewhere [13]. The reported analyses therefore explored the feasibility of using data from EPRs to evaluate outcome measures for intervention trials of stroke prevention.

## Methods

### Participants

Data for this study were derived from the UK General Practice Research Database (GPRD). The GPRD is a large database that contains electronic patient records from over 400 family practices in the UK. The geographic distribution and demographic profile of the GPRD are representative of the UK population [14]. The validity of medical diagnoses and prescribing information in GPRD has been confirmed in several studies [15]. In the GPRD, data are designated as 'up-to-standard' (UTS) when they are judged to be of high enough quality to be used for research.

Data for the present analyses were extracted from the records of 414 UK general practices with a registered population of approximately 3.2 million in 2006. The initial sample comprised 48,239 participants. The last data were collected in August 2007. Stroke patients were identified using the 202 READ and OXMIS codes for stroke that were described previously [16,17]. Transient ischemic attacks were not included. A first stroke was defined as a first diagnosis of stroke during the period 01/01/1997-31/12/2006 inclusive with a minimum of 24 months of up-to-standard record free from stroke codes preceding the index event. This ensured that all stroke patients represent new diagnoses. Incidence and case-fatality rates for stroke in GPRD appear to be comparable to estimates obtained from other epidemiological studies [17].

For the present analyses, only those individuals for whom a first stroke event was recorded between 1st January 2003 and 31st December 2006 were retained because these represented recent years' data with improved quality data recording and greater relevance to contemporary clinical practice. Data for 64 individuals with stroke diagnoses first recorded after the death date were excluded. The final study sample comprised 21,330 participants with a first stroke event between 1st January 2003 and 31st December 2006. The mean age of the participants was 73 years and 53% were women.

### Data for analysis

The electronic patient record was evaluated for each subject for the 12 months preceding, and for the 12 months after, the stroke index date. The stroke index date was defined as the first date on which a stroke medical code was recorded. Data were analysed for six continuous variables: systolic blood pressure (BP; mm Hg); diastolic BP (mm Hg); total cholesterol (mmol/l); low density lipoprotein (LDL) cholesterol (mmol/L); high density lipoprotein (HDL) cholesterol (mmol/L); and body mass index (BMI; Kg/m^2^). For each of the continuous measures, the mean of all values recorded in each participant in the 12 months before, or after, stroke was estimated.

Electronic patient records were also evaluated for a further ten dichotomous outcomes including whether: the blood pressure was recorded; antihypertensive drugs were prescribed; cholesterol was measured (including total, LDL or HDL cholesterol); statins were prescribed; atrial fibrillation was recorded; antiplatelet drugs were prescribed; whether anticoagulants were prescribed; whether the body mass index was recorded; whether smoking or alcohol habits were recorded. These outcomes were extracted from the clinical, referral and therapy files using READ medical codes or BNF formulary codes.

Recording of blood pressure, cholesterol, atrial fibrillation, and body mass index (BMI) were characterised by whether or not one or more measurements were recorded in either the year before or the year after stroke. Drug prescriptions were analysed in the same way. Antihypertensive drugs included ACE inhibitors, angiotensin-II receptors, calcium-channel blockers, centrally acting antihypertensive drugs, diuretics, beta-blockers, and adrenergic neurone blocking drugs. Antiplatelet drugs included aspirin and dipyridamole. Anticoagulants included oral (e.g. warfarin) anticoagulants and just a small number of parenteral (e.g. heparin) anticoagulant prescriptions. Information concerning smoking included whether the participant's smoking habit was recorded, as well as whether the individual was recorded as a current smoker in the 12 months. Information concerning alcohol included whether the alcohol intake was recorded, and whether alcohol intake was higher than recommended limits. Recommended upper limits were 21 units per week for men and 14 units per week for women.

### Statistical analysis

For binary outcomes, because of right censoring following stroke arising from early mortality, a series of time-to-event analyses were implemented to estimate the proportion of stroke participants with the outcomes of interest during the 12 months following the stroke index date. For continuous measures, the study investigated the mean values for systolic and diastolic blood pressure, cholesterol levels (total, LDL, and HDL), and BMI values in the 12 months before and after the stroke index date. For each measure, the participant-specific mean was estimated by stroke index year. For lifestyle measures, the study estimated the proportion of stroke participants that, at any time during the 12 months before or after the stroke index date, were recorded as current smokers or consuming over the recommended units of alcohol per week. For participants with outcome data available both before and after the stroke index date, a random-effects model FOR REPEATED MEASURES, with participant as the random effect, was used to estimate gender and age adjusted differences (and associated confidence intervals). For the binary measures (smoking and alcohol) a two-sample proportion test was used to examine differences (and associated confidence intervals) in the proportions before and after stroke.

Additional analyses were carried out to explore the variation in the distribution of outcome measures at different family practices. The intraclass correlation coefficient (ICC) by family practice was calculated using one-way ANOVA for the continuous outcome measures: systolic and diastolic blood pressure, cholesterol, and BMI index. All analyses were carried out using STATA version 11.

#### Ethics

The study was implemented utilising an anonymised dataset from the General Practice Research Database. The study protocol was approved by the Independent Scientific Advisory Committee (ISAC) of the Medicines and Healthcare products Regulatory Agency (MHRA) (ISAC Protocol No. 07_027R).

## Results

In the 4-year study period from 1st January 2003 to 31st December 2006, a total of 22,730 first-ever stroke diagnoses were recorded in the GPRD. The frequency of records of blood pressure, cholesterol, BMI, and lifestyle factors are presented in Table 1. Risk factor recording was more frequent after stroke than before. In the 12 months following stroke, BP was recorded for 90%, cholesterol for 70%, BMI for 47% and smoking and alcohol habits for 73% and 35% respectively. Risk factor management was greater after stroke than before, and generally increased over time, with between two thirds and three quarters of patients being treated with antihypertensive drugs or statins. A smaller proportion (7%) of patients had a record of anticoagulant prescription in the 12 months before the stroke index date which increased to12%in the 12 months after the stroke index date. Low anticoagulant prescriptions appeared to be consistent with the small proportion of patients recorded as having atrial fibrillation in the period. A third (33%) of patients with atrial fibrillation were treated with anticoagulants in the 12 months before the stroke index date compared to 55% in the 12 months after the stroke index date.

**Table 1:** Primary care management in the 12 months before and after stroke by year of stroke.

		2003	2004	2005	2006	Total
		(N = 5,334)	(N = 6,248)	(N = 5,764)	(N = 5,384)	(N = 22,730)
						
**BP record**	Before stroke	3,453 (70%)	4,539 (77%)	4,320 (80%)	4,137 (81%)	16,449 (77%)
	After strok**e**^**a**^	3,813 (88%)	5,026 (93%)	4,602 (93%)	4,183 (87%)	17,624 (90%)
						
**Antihypertensive drugs prescribed**	Before stroke	2,798 (57%)	3,544 (60%)	3,358 (62%)	3,167 (62%)	12,867 (60%)
	After stroke ^**a**^	3,118 (69%)	3,891 (71%)	3,677 (73%)	3,464 (72%)	14,150 (71%)
						
**Antiplatelet drugs**	Before stroke	1,920 (39%)	2,600 (44%)	2,475 (46%)	2,321 (46%)	9,316 (44%)
**prescribed**	After stroke ^**a**^	2,865 (64%)	3,546 (65%)	3,301 (65%)	3,074 (64%)	12,786 (65%)
						
**Anticoagulants**	Before stroke	270 (6%)	364 (6%)	402 (7%)	355 (7%)	1,391 (7%)
**prescribed**	After stroke ^**a**^	444 (10%)	603 (11%)	627 (13%)	557 (12%)	2,231 (12%)
						
**Atrial fibrillation**	Before stroke	139(3%)	179(3%)	185(3%)	187(4%)	690(3%)
	After stroke ^**a**^	238(5%)	285(5%)	265(5%)	267(6%)	1055(5%)
						
**Cholesterol recording (Total, LDL or HDL)**	Before stroke	1,724 (35%)	2,828 (48%)	2,970 (55%)	2,833 (56%)	10,355 (49%)
	After stroke ^**a**^	2,691 (64%)	3,955 (76%)	3,681 (76%)	2,921 (62%)	13,248 (70%)
						
**Statin prescription**	Before stroke	1,042 (21%)	1,799 (31%)	1,941 (36%)	2,038 (40%)	6,820 (32%)
	After stroke ^**a**^	2,499 (56%)	3,554 (65%)	3,485 (69%)	3,433 (71%)	12,971 (65%)
						
**BMI record**	Before stroke	1,391 (28%)	2,050 (35%)	1,953 (36%)	2,042 (40%)	7,436 (35%)
	After stroke ^**a**^	2,249 (39%)	2,417 (48%)	1,959 (54%)	2,353 (46%)	8,978 (47%)
						
**Smoking record**	Before stroke	1,633 (33%)	3,199 (54%)	3,115 (57%)	2,841 (56%)	10,788 (51%)
	After stroke ^**a**^	2,783 (66%)	4,176 (79%)	3,829 (79%)	3,262 (68%)	14,050 (73%)
						
**Alcohol record**	Before stroke	972 (20%)	1,596 (27%)	1,343 (25%)	1,3222 (26%)	5,233 (25%)
	After stroke ^**a**^	1,356 (32%)	1,982 (37%)	1,998 (40%)	1,556 (32%)	6,892 (35%)

Figures are frequencies (column percents).

^a ^'After stroke' figures are estimated from time-to- event analyses

Table 2 describes substantial variation in adherence to stroke secondary prevention recommendations among family practices. In general the proportion of patients with measures recorded ranged from 0 to 100% at different practices. Mean values for continuous measures and intraclass correlation coefficients for the main outcome measures by family practice are presented in Table 3. The intraclass correlation coefficient for the systolic BP in the 12 months before stroke was 0.02 and 0.03 in the 12 months after stroke. For diastolic BP, the intraclass correlation coefficient was 0.03, and for both BMI and total cholesterol 0.02, both before and after stroke. A higher intraclass correlation coefficient of 0.05 was observed for both LDL and HDL cholesterol both before and after stroke.

**Table 2:** Centiles for family practice-specific proportions (%) of stroke secondary prevention measures

		Lowest performing family practice	Lower quartile	Median	Upper quartile	Highest performing family practice
						
**BP recorded**	Before stroke	33	71	77	83	100
	After strok**e**	0	76	82	87	100
						
**Antihypertensive treatment**	Before stroke	0	67	73	79	100
	After strok**e**	0	77	81	86	100
						
**Antiplatelet treatment**	Before stroke	0	34	43	50	100
	After strok**e**	0	53	59	65	100
						
**Anticoagulant treatment**	Before stroke	0	3	6	9	25
	After strok**e**	0	7	10	14	50
						
**Atrial fibrillation**	Before stroke	0	0	3	5	25
	After strok**e**	0	2	5	7	43
						
**Cholesterol recorded**	Before stroke	0	38	46	56	100
	After strok**e**	0	53	61	69	88
						
**Statin measure**	Before stroke	0	23	30	38	100
	After strok**e**	0	53	60	68	100
						
**BMI recorded**	Before stroke	0	25	33	44	100
	After strok**e**	0	26	37	52	83
						
**Smoking recorded**	Before stroke	0	41	50	59	100
	After strok**e**	0	55	63	72	100
						
**Alcohol recorded**	Before stroke	0	14	22	34	100
	After strok**e**	0	12	25	44	80

**Table 3:** Descriptive statistics and intraclass correlation coefficient (ICC) by family practice for continuous measures

		N	Mean (SD)	ICC (SE)
				
**SBP measure(mmHg)**	Before stroke	16,462	144.76(19.13)	0.020 (0.003)
	After stroke	17,626	139.08(17.05)	0.025 (0.004)
				
**DBP measure(mmHG)**	Before stroke	16,462	80.24(10.12)	0.028 (0.003)
	After stroke	17,624	78.02 (9.18)	0.028 (0.004)
				
**Total cholesterol(mmol/l)**	Before stroke	10,318	5.10(1.16)	0.018 (0.004)
	After stroke	13,210	4.60(1.06)	0.021 (0.004)
				
**LDL cholesterol(mmol/l)**	Before stroke	5744	2.93(1.03)	0.047 (0.009)
	After stroke	7,402	2.49(0.91)	0.054 (0.008)
				
**HDL cholesterol(mmol/l)**	Before stroke	7,305	1.44(0.44)	0.045 (0.007)
	After stroke	9,182	1.44(0.44)	0.052 (0.007)
				
**BMI measure(kg/m**^**2**^**)**	Before stroke	6,958	27.21(5.47)	0.018 (0.005)
	After stroke	8,547	26.92(5.25)	0.016 (0.004)

Table 4 presents the results based on patients who were selected because their records provided information both before and after the stroke event. There were quantitatively important reductions from before- to after-stroke in systolic and diastolic blood pressure as well as total and LDL cholesterol, BMI, current smoking and excessive drinking. Systolic BP was 6mm Hg lower after stroke than before, total cholesterol was 0.6 mmol/l lower after stroke than before, LDL cholesterol was 0.7 mmol/l lower, BMI was 0.3 kg/m^2 ^lower, and there was a reduction in the proportion of active smokers of 5% and excessive drinkers of 4%. There was also evidence of a declining secular trend in values for systolic and diastolic blood pressure, as well as for total and LDL cholesterol. Tests for trend gave P values of 0.004 or smaller for each of these measures either before or after stroke.

**Table 4:** Mean values for stroke prevention measures for participants with values recorded both before and after stroke.

		2003	2004	2005	2006	Overall
						
**Systolic BP **(N = 14,006)	Before stroke	148.64	145.72	144.49	143.31	145.54
	After stroke	141.60	139.28	139.24	137.78	139.48
	Difference( 95% CI)	-7.04(-7.69;-6.39)	-6.44(-6.97;-5.90)	-5.25(-5.79;-4.70)	-5.53(-6.10;-4.95)	-6.02(-6.03;-6.01)
						
**Diastolic BP **(N = 14,006)	Before stroke	82.21	80.58	80.17	79.60	80.64
	After stroke	78.90	77.70	77.54	77.18	77.83
	Difference( 95% CI)**	-3.31(-3.66;-2.96)	-2.88(-3.18;-2.59)	-2.63(-2.93;-2.32)	-2.43(-2.75;-2.11)	-2.78(-2.79;-2.77)
						
**Total cholesterol **(N = 7,085)	Before stroke	5.38	5.28	5.09	4.96	5.18
	After stroke	4.65	4.52	4.41	4.36	4.49
	Difference( 95% CI)**	-0.73(-0.79;-0.67)	-0.76(-0.80;-0.71)	-0.69(-0.73;-0.64)	-0.60(-0.65;-0.55)	-0.60(-0.61;-0.59)
						
**LDL cholesterol **(N = 3,206)	Before stroke	3.11	3.12	2.97	2.77	2.99
	After stroke	2.44	2.40	2.33	2.30	2.38
	Difference( 95% CI)**	-0.67(-0.76;-0.57)	-0.73(-0.79;-0.66)	-0.63(-0.69;-0.57)	-0.47(-0.53;-0.40)	-0.70(-0.71;-0.69)
						
**HDL cholesterol **(N = 4,287)	Before stroke	1.40	1.44	1.45	1.44	1.43
	After stroke	1.39	1.44	1.44	1.41	1.42
	Difference( 95% CI)	-0.02(-0.04;0.01)	-0.01(-0.02;0.01)	-0.01(-0.02;0.00)	-0.03(-0.04;-0.02)	-0.02(-0.02;-0.01)
						
**BMI values **(N = 3,881)	Before stroke	27.83	27.86	27.56	27.87	27.78
	After stroke	27.52	27.50	27.17	27.58	27.44
	Difference( 95% CI)	-0.31(-0.45;-0.17)	-0.37(-0.48;-0.25)	-0.39(-0.51;-0.26)	-0.30(-0.41;-0.18)	-0.34(-0.34;-0.33)
						
**Current smokers **(N = 7,213)	Before stroke	31%	26%	24%	27%	26%
	After stroke	23%	21%	21%	21%	21%
	Difference( 95% CI)	-8%(-12%;-4%)	-5%(-7%;-2%)	-3%(-6%;-1%)	-6%(-9%;-3%)	-5%(-6%;-3%)
						
**Excessive drinking **(n = 1,968)	Before stroke	13%	12%	12%	13%	12%
	After stroke	8%	7%	9%	10%	8%
	Difference( 95% CI)	-5%(-10%1%)	-5%(-9%;-1%)	-3%(-7%1%)	-4%(-9%;1%)	-4%(-6%;-2%)

Figures are means except where indicated.

Table 5 presents the proportion of patients who achieved recommended target values for the measures analysed. For each measure, the denominator was the total with the item recorded. Between one quarter to a third of patients achieved systolic BP target, but the proportion was almost two-thirds for diastolic BP. Just under a half of patients achieved total cholesterol targets while just over half of stroke patients achieved LDL cholesterol targets. A similar finding emerged with respect to BMI values. More encouraging results emerged with respect to HDL cholesterol, smoking, and alcohol units per week where less than a tenth to around a fifth of the patients with values recorded were outside the recommended guidelines.

**Table 5:** Proportion of stroke patients with values recorded who were within the range recommended by the Intercollegiate Stroke Working Party guidelines in the 12 months following a stroke index date

		N	2003	2004	2005	2006	Overall
							
**Blood pressure**	Systolic (< 130 mmHg)	14,006	26%	29%	29%	33%	29%
	Diastolic (< 80 mmHg)	14,006	58%	63%	64%	66%	63%
							
**Cholesterol**	Total (< 4 mmol/l or -25%)	7,085	41%	42%	47%	49%	47%
	LDL (< 2 mmol/l or -30%)	3,206	51%	50%	53%	51%	51%
	HDL (> 1.00 mmol/l)	4,287	89%	90%	89%	87%	89%
							
**Body Mass Index**	Weight loss (kg/m^2^)	3,881	53%	55%	55%	54%	54%
							
**Smoking**	Non-smokers	7,213	77%	79%	79%	79%	79%
							
**Alcohol units per week**	Men = < 21;women < 14	1,968	92%	93%	91%	90%	92%
							

## Discussion

### Main findings

The present results show that electronic patient records from primary care provide a valuable tool for evaluating vascular risk factor values including blood pressure, cholesterol, BMI and smoking. Data on drug prescriptions provide the opportunity to assess adherence with guidelines in secondary stroke prevention. The data presented may be used to inform the design of stroke prevention studies in primary care.

This study represents one of the first primary care-based stroke studies to compare risk factor values from before to after stroke. Among participants with values available both before and after stroke, there were clinically important overall reductions in blood pressure, cholesterol, BMI, cigarette smoking and excessive drinking from before to after stroke. These differences appeared to diminish slightly over time, possibly as a result of a declining secular trend in pre-stroke risk factor values. Reductions in BMI may be favourable but might sometimes be a sign of impaired nutrition after stroke. Increased prescribing of antihypertensive drugs, statins after stroke may have contributed to observed reductions in blood pressure and cholesterol values. These might potentially translate into better outcomes for stroke patients, including survival and reduced recurrence rates [8,10,11]. The substantial variation in drug prescriptions at a practice level reinforce previous findings [18] and extend these findings to GP consultations. Several explanations may be offered for the observed between-practice variation including practitioner-level factors, including the acceptability of guidelines, and practice-level factors including practice management and the practice population. These suggestions could not be tested here but need considering when designing cluster-level interventions to improve adherence to recommended guidelines.

### Comparison with earlier studies

The present study findings are consistent with previous population-based studies that found recent declines in the proportion of smokers, as well as in mean total cholesterol levels, and mean systolic and diastolic blood pressure in stroke patients [19,20]. The study findings on the proportion of stroke participants treated with anticoagulants and antiplatelet drugs following stroke is consistent with previous studies [3,21-23]. Grau et al. [21] reported that antiplatelet drugs were administered to 71% of their sample. Rudd et al. [3] found that 64% of stroke participants had a cholesterol measure available following stroke with about 63% prescribed a statin. Results for the proportion of participants on anti-hypertensive treatment are similar to those reported previously [3,17,22,23]. Given sampling difference as well as variation in the follow-up period length between studies may explain modest variations in figures.

### What this study adds

Can data from primary care electronic records inform the evaluation of stroke secondary prevention in primary care? The findings of this study suggest that GPRD data represent potentially a valuable resource to evaluate whether adherence to recommended stroke prevention guidelines could improve stroke prognosis. However, this potential value must be qualified in several respects. Several aspects of stroke management are specific to stroke subtypes. However, most strokes are recorded in GPRD using codes that do not distinguish between haemorrhage and infarction. More detailed classification of stroke types using information from CAT or MRI scans is not usually feasible. A further concern is the low level of recording of lifestyle factors recording, in particular obesity, smoking and alcohol consumption. As a consequence we cannot be confident that our evaluation of the extent to which the recommended guidelines regarding these factors is generalisable to other stroke patients. Moreover, the lack of additional socio-demographic data places constraints on the ability to verify that missing data on different outcome measures is ignorable.

This study provides new evidence on the magnitude of risk factor reductions during the 12 months following index stroke. Against a background of declining secular trends in values for blood pressure and cholesterol, this study showed that there were important reductions in blood pressure, cholesterol, body mass index, cigarette smoking and excessive alcohol drinking from before to after stroke (2006 data is incomplete because a 6-month delay in data collection from practices by the GPRD). Epidemiologic studies suggest that even modest reductions in systolic blood pressure of 5-6 mmHg, or diastolic of 2-3mm Hg, may be associated with a 20% to 28% reduced risk of stroke [24,25]. The reduction in cholesterol observed is also quantitatively important in the context of evidence from clinical trials of cholesterol lowering therapy [11]. Reductions in BMI may be regarded favourably but may also be indicative of nutritional problems following stroke. Little evidence is available concerning trends in smoking and alcohol use in stroke populations, but there appear to be important reductions following stroke. However, the present results emphasise the need for improved data recording with respect to these risk factors for stroke recurrence. This is important considering the potential value of EPRs for health care research and policy development. The study also documents important practice level variations in the proportion of stroke participants with records or treatment for several risk factors. The factors underlying practice level variation could further help the implementation of the guidelines for secondary stroke prevention, an outcome that will be addressed through our research.

### Limitations of this study

This study had the strength of a large sample drawn from a large number of family practices from across the UK. However, several limitations of the data must be acknowledged. It was not generally possible to distinguish sub-types of stroke. Only 17% of strokes could be classified as either haemorrhages or infarctions. The stroke type has relevance for certain aspects of secondary prevention including the prescriptions of statins, aspirin and antiplatelet drugs, and anticoagulants. However, lowering BP has been shown to reduce the risk of both ischemic and hemorrhagic stroke, while smoking and alcohol consumption are also associated with risk of stroke [20,26,27]. Also, while all stroke diagnoses were recorded prospectively by practices, stroke patients may be admitted directly to hospital and their first contact with the practice may be for review at some later date. The stroke onset date may be therefore imprecisely recorded in primary care, perhaps often being recorded after the true stroke date. However, the validity of clinical data included in the GPRD has repeatedly been shown to be high, and data are rigorously checked and regularly audited. Another limitation of this research is the lack of a reference method for stroke diagnosis as discussed elsewhere [16]. It is also important to acknowledge that, for an appreciable number of participants, information about their outcome measures was not available. The study findings are comparable, however, to those from previous studies [3,28,29]. Stroke secondary prevention was evaluated with respect to the most recent UK guidelines which represent a 2008 update of the 2004 edition. Trends showed some lack of consistency between 2005 and 2006. This is probably explained by the shorter duration of follow-up for some participants with a stroke index date 2006 compared to those with a stroke during previous years.

## Conclusions

The present study has documented, in a large nationally representative sample, that electronic based patient records represents potentially a valuable source for evaluating guidelines implementation with respect to secondary stroke prevention. The broad nature of the GPRD data allowed us to identify and operationalise several outcome measures stipulated in the ICSWP as important targets for secondary stroke prevention. The study findings indicate a consistent increase in the screening and treatment of stroke patients over time, but also the need for further improvement both in screening and drug prescriptions. The overall message of the study is that GPRD data is suitable for the implementation of cluster randomised trials to evaluate the success or failure of health interventions on stroke patients' outcomes.

## Competing interests

The authors declare that they have no competing interests.

## Authors' contributions

AD and MG conceptualised the paper worked on the data set, conducted the analyses, and wrote of the paper. MG, AMT, CW, AR, and MA acquired the permits and data set and provided feedback on the paper. All authors read and approved the final manuscript.

## Pre-publication history

The pre-publication history for this paper can be accessed here:

http://www.biomedcentral.com/1471-2458/11/86/prepub
